# Surges of hospital-based rhinovirus infection during the 2020 coronavirus disease-19 (COVID-19) pandemic in Beijing, China

**DOI:** 10.1007/s12519-021-00477-2

**Published:** 2021-10-28

**Authors:** Ruo-Xi Zhang, Dong-Mei Chen, Yuan Qian, Yu Sun, Ru-Nan Zhu, Fang Wang, Ya-Xin Ding, Qi Guo, Yu-Tong Zhou, Dong Qu, Ling Cao, Chun-Mei Zhu, Lin-Qing Zhao

**Affiliations:** 1 Laboratory of Virology, Beijing Key Laboratory of Etiology of Viral Diseases in Children, Capital Institute of Pediatrics, Beijing, 100020 China; 2 Intensive Care Unit, Affiliated Children’s Hospital, Capital Institute of Pediatrics, Beijing, 100020 China; 3 Department of Respiratory, Affiliated Children’s Hospital, Capital Institute of Pediatrics, Beijing, 100020 China

**Keywords:** Children, Coronavirus disease-19 (COVID-19), Human rhinovirus, Preventive measures, Respiratory viruses

## Abstract

**Background:**

A series of public health preventive measures has been widely implemented in Beijing to control the coronavirus disease-19 (COVID-19) pandemic since January 2020. An evaluation of the effects of these preventive measures on the spread of other respiratory viruses is necessary.

**Methods:**

Respiratory specimens collected from children with acute respiratory infections were tested by NxTAG™ respiratory pathogen panel assays during January 2017 and December 2020. Specimens characterized as rhinoviruses (RVs) were sequenced to identify the RV species and types. Then, the epidemiology results of respiratory pathogens in 2020 were compared with those from 2017 to 2019 using SPSS statistics 22.0.

**Results:**

The positive rates of adenovirus (ADV), influenza virus (flu), RVs, and respiratory syncytial virus (RSV) dropped abruptly by 86.31%, 94.67%, 94.59%, and 92.17%, respectively, from February to May 2020, compared with the average level in the same period during 2017–2019. Positive rates of RVs then steeply increased from June 2020 (13.77%), to an apex (37.25%) in August 2020, significantly higher than the average rates (22.51%) in August 2017–2019 (*P* = 0.005). The increase, especially in group ≥ 3 years, was accompanied by the reopening of schools and kindergartens after the 23rd and 24th week of 2020 in Beijing.

**Conclusions:**

Whereas the abrupt drop in viral pathogen positive rates from February to May 2020 revealed the remarkable effects of the COVID-19 preventive measures, the sharp increase in positive rates of RVs from the 23rd week of 2020 might be explained by the reopening of schools and kindergartens in Beijing.

**Supplementary Information:**

The online version contains supplementary material available at 10.1007/s12519-021-00477-2.

## Introduction

The coronavirus disease-19 (COVID-19) pandemic has had a profound and disastrous impact on society since 2020. To control the transmission of the disease, a series of public health preventive measures has been widely implemented since January 2020, including social withdrawal, school closures, wearing masks, travel restrictions, personal hygiene improvements, and border closures in Beijing, China. The start of the COVID-19 restriction measures in the northern hemisphere coincides with the winter influenza virus (Flu) and respiratory syncytial virus (RSV) seasons, and some reports indicate that the local Flu and RSV seasons ended early at the same time [1]. In the southern hemisphere, the data from Oceania (Australia), South America (Chile), and Southern Africa (South Africa) showed very low flu activity from June to August 2020, the months that constitute the typical southern hemisphere flu season [2]. However, Australian researchers found that the detection rate of human rhinovirus (RV) was much higher than the average from the 22nd week, which is completely different from the reduction trend of most respiratory viruses [3]. Therefore, we were curious about the effects of these COVID-19 preventive measures on the spread of other respiratory viruses, such as human respiratory syncytial virus (RSV), influenza virus (flu) A and B, adenovirus (ADV), and human rhinovirus (RV), the most important causes of acute respiratory viral infections leading to morbidity and mortality worldwide especially in vulnerable individuals [4].

Beijing, the capital of the People’s Republic of China, is located at the northern part of the North China Plain and has a semi-humid continental climate in the warm temperate zone. In previous studies, the epidemic season of RSV in Beijing has been identified from October in each year to March of the following year [5]. At a global level, RV infection occurs throughout the entire year, with peaks in autumn and winter in temperate regions [6]. In temperate climates, flu transmission has an obvious seasonal pattern. Winter is the season for flu spread in northern China, and cold weather and dry air will increase its incidence [7]. ADV can be detected throughout the year in Beijing; however, its prevalence is obviously related to the season, and the incidence rate is higher in summer [8].

It has been reported that viral interference among the flu virus, RVs, and other respiratory viruses can affect viral infections at both the host and population levels [9]. To investigate the effect of the COVID-19 pandemic and associated preventive measures on the epidemiology of common respiratory virus, especially RV, we retrospectively analyzed the epidemiological characteristics of viral pathogens in clinical specimens collected from children with acute respiratory infections in Beijing from January to December 2020 and compared them to those from January 2017 to December 2019. This will help to improve the preventive measures for viral infections in children.

## Methods

### Clinical specimens

Respiratory specimens (including nasal swabs, throat swabs, nasopharyngeal aspirates, or sputum) were collected from pediatric patients under 14 years of age. These patients were diagnosed with acute respiratory infections at the Affiliated Children’s Hospital, Capital Institute of Pediatrics (Beijing, China) from January 2017 to December 2020. These patients were then tested for multiple respiratory pathogens via screening.

Upon arrival at the laboratory, clinical specimens were handled in a Class II biosafety cabinet and were processed immediately using 2.5 mL of viral transport medium (Yocon Biotechnology Co., Ltd, Beijing, China). The specimens were then centrifuged (500×*g*, 10 min) to obtain the supernatant for multiple respiratory pathogen screening. The study was approved by the Ethics Committee of the Capital Institute of Pediatrics, China (SHERLLM2019005).

### NxTAG™ respiratory pathogen panel assays

Total nucleic acid (DNA and RNA) was extracted from 200 µL of each collected specimen using the QIAamp MinElute Virus Spin Kit (Qiagen GmbH, Germany) according to the manufacturer’s instructions. The MS-2 bacteriophage was used as an internal control. Nucleic acid extracts were tested for flu, RSV, parainfluenza virus (PIV), ADV, human metapneumovirus (HMPV), human coronaviruses (HCoVs, such as 229E, NL63, OC43, and HKU1), enterovirus (EV)/RVs, human bocavirus (HBoV), *Mycoplasma pneumonia* (Mpneu), and *Chlamydia pneumoniae* (Cpneu) using NxTAG™ respiratory pathogen panel (RPP) assays (Luminex Molecular Diagnostics Inc., Toronto, Canada) in a 96-well plate format according to the manufacturer’s instructions. The plate was then analyzed using Luminex xPONENT, and the resultant data were analyzed using Luminex SYNCT Data Analysis Software (Luminex). A mean fluorescence intensity value above the threshold level for a particular target indicated a positive result for that target.

### Identification and genotyping of rhinoviruses using reverse-transcriptase polymerase chain reaction and sequence analysis

Clinical specimens positive for EVs/RVs were subjected to semi-nested reverse-transcriptase polymerase chain reaction (RT-PCR) for RV confirmation by obtaining a 539-bp fragment that targets the *VP4/VP2* gene region [10]. For RV-negative specimens, RT-PCR was performed to screen for EVs [11]. All PCR products were sequenced by Sino Geno Max Co., Ltd. (Beijing, China).

Sequences were verified using NCBI BLAST (http://blast.ncbi.nlm.nih.gov/). RV or EV species were identified via phylogenetic analyses of sequences using MEGA version 6.0, using the neighbor-joining method. To build phylogenetic trees, bootstrap values were estimated with 1000 replications to assess the reliability of each individual node [12].

### Clinical data collection

The medical records of pediatric patients with confirmed infection of RV species were reviewed. The following clinical data were extracted from the records: age, gender, length of hospital stay, sample collection date, clinical diagnosis, and laboratory values.

### Statistical analysis

Because the ages of the RV-positive patients and length of hospital stay were not normally distributed, they were described here using the median and interquartile ranges. Chi-square (*χ*^2^) and rank sum tests were used for statistical analysis with SPSS Statistics (version 22.0, IBM, NY, USA). Statistical significance was set at *P* < 0.05.

## Results

### Multiple respiratory pathogenic screening using NxTAG™ respiratory pathogen panel assays

From January 2017 to December 2020, 7434 respiratory specimens were collected for respiratory virus screening using NxTAG™ RPP assays. Patients with several specimens in one hospitalization or hospital visit had only the first specimen retained. Moreover, patients older than 14 years of age were excluded. Only 6689 specimens were included into this analysis after these exclusions. Among these specimens, 815 (12.18%, 815/6689) were positive for RSV, 223 (3.33%, 223/6689) for flu, 519 (7.76%, 519/6689) for PIV, 204 (3.05%, 204/6689) for HMPV, 317 (4.74%, 317/6689) for ADV, 998 (14.92%, 998/6689) for EVs/RVs, 455 (6.80%, 455/6689) for HBoV, 135 (2.02%, 135/6689) for HCoV, 773 (11.56%, 773/6689) for Mpneu, and 34 (0.51%, 34/6689) for Cpneu.

**Fig. 1:**
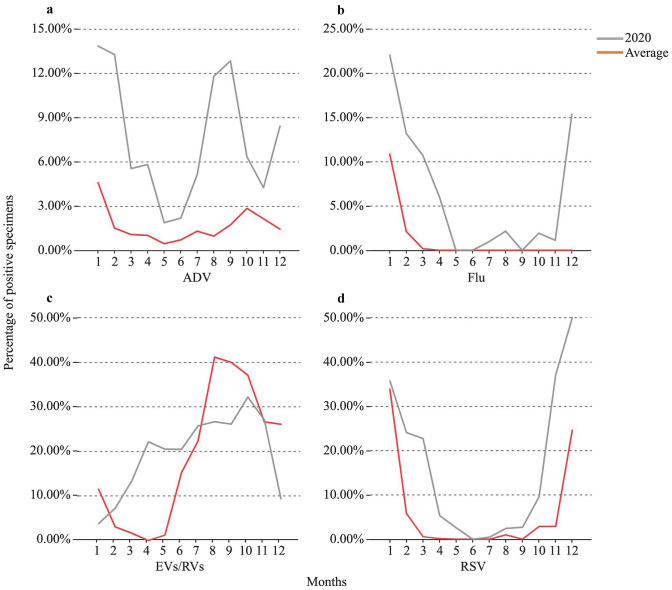
Comparison of monthly distribution of several respiratory viruses (not all included) screened in the study based on the percentage of positive specimens in 2020 and the average during Jan 2017–Dec 2019. **a** The percentage of adenovirus (ADV)-positive specimens relative to the number of tests in 2020 (red curve) compared with the average value in 2017–2019 (grey curve); **b** the percentage of influenza (Flu)-positive specimens relative to the number of tests in 2020 (red curve) compared with the average value in 2017–2019 (grey curve); **c** the percentage of enterovirus (EV)/rhinoviruses (RV)-positive specimens relative to the number of tests in 2020 (red curve) compared with the average value in 2017–2019 (grey curve); **d** the percentage of respiratory syncytial virus (RSV)-positive specimens relative to the number of tests in 2020 (red curve) compared with the average value in 2017–2019 (grey curve)

Compared with the average level in the same periods of 2017–2019, the positive rates of ADV, flu, EVs/RVs, and RSV dropped by 86.31%, 94.67%, 93.13%, and 92.17%, respectively (*P* < 0.001), and the remained below 6.00%, from February 2020 to May 2020 (Fig. 1). In particular, the virus positive rates of the 2019–2020 flu and RSV seasons had dropped to 0.00%. However, the positive rate of EVs/RVs ascended in a steep curve from 15.22% in June 2020, to its apex of 41.18% in August 2020, which was significantly higher than the average rates (26.41%) in August for the period 2017–2019 (*P* = 0.007). The positive rates of other viruses remained below 2.00%.

### Molecular epidemiology of RVs

Among 998 specimens positive for EVs/RVs, 892 (89.38%, 892/998) were RV-positive, whereas 27 (2.71%, 27/998) were EV positive and 79 were undetermined with low-yield amplification products.

**Fig. 2:**
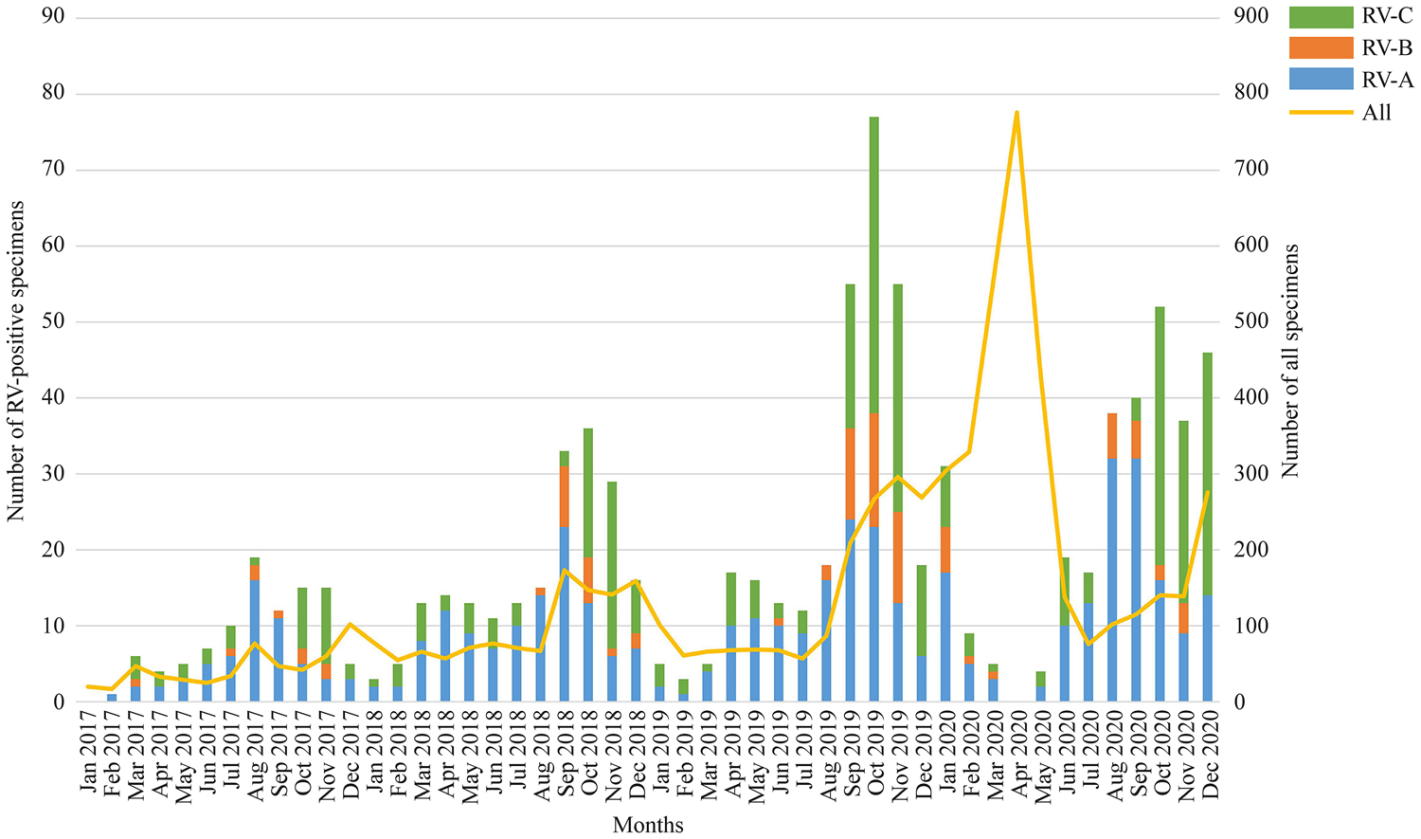
Monthly distribution of rhinoviruses (RVs) from Jan 2017 to Dec 2020. The stacked column charts correspond to the left axis, indicating the number of positive specimens of various species of RVs in each month; the line graph corresponds to the right axis, indicating the number of specimens screened by the NxTAG™ RPP assays in each month

From 2017 to 2019, RV infections occurred year round with 75.11% detected in the autumn and winter months (Fig. 2). In 2020, the positive rate of RV infection decreased by 94.59% from February to May, even though the number of cases for screening increased by 71.46%. Subsequently, the positive rates of RVs steeply increased from June 2020 (13.77%), until reaching its apex (37.25%) in August 2020. The apex was significantly higher than the average August rates (22.51%) in 2017–2019 (*P* = 0.005).

Patients positive for RVs in 2020 with median age 2.351 years (interquartile range: 0.859–4.248 years) were significantly older than those in 2017–2019 (median age: 1.062 years, interquartile range: 0.342–3.425 years, *P* < 0.001). However, the length of hospital stay days showed no significant difference with those in 2017–2019 (*P* = 0.871).

Among 892 specimens positive for RVs, 452 (50.67%, 452/892) were determined as RV-A, 94 (10.54%, 94/892) for RV-B, and 346 (38.79%, 346/892) for RV-C. In the present study, RV-A and RV-B were the dominant species during August and September, accounting for 73.04% and 16.09%, whereas RV-C, accounting for 59.10%, became the dominant species from October to December. The monthly distribution of RV species in 2020 was not significantly different from that during 2017–2019 (*P* = 0.324). Clinical information revealed that the proportions of male children with RV-A, RV-B, and RV-C were 64.4%, 47.9%, and 61.6%, respectively (Table 1). In all 4 years, children positive for RV-A had a median age of 1.215 years (interquartile range: 0.379–3.425 years) and were younger than those positive for RV-C (median age: 1.706 years, interquartile range: 0.597–3.698 years, *P* = 0.03).

**Table 1:** Clinical information for children positive for different species rhinoviruses (RVs) during 2017–2020

	2017–2019			2020		
Variables	RV-A	RV-B	RV-C	RV-A	RV-B	RV-C
Number	299	69	226	153	25	120
Male	191 (63.9%)	33 (47.8%)	139 (57.1%)	100 (65.4%)	12 (48.0%)	74 (61.7%)
Age of patients (years)^a^	0.789 (0.228–2.657)	1.632 (0.397–6.139)	1.357 (0.489–3.567)	2.504 (0.990–4.646)	1.017 (0.386–5.432)	2.073 (0.845–3.821)
The length of hospital stay (days)^a^	7 (3–12)	7 (5–12.75)	7 (5–13)	7 (3–12)	6 (4–17)	6 (5–11)

^a^Described using the median and interquartile ranges

In the phylogenetic trees constructed with MEGA 6.0 software (Supplementary Fig. 1), 452 specimens positive for RV-A were grouped into 62 types, whilst, 94 specimens positive for RV-B were grouped into 19 types, and 346 specimens positive for RV-C were grouped into 42 types. The dominant types of RV-A were RV-A101, A11, A47, and A44 in 2020 RV-A78 and RV-A12 in 2019, RV-A24 in 2018, and RV-A49 in 2017. For RV-B and RV-C, the most common types were RV-B83 and RV-C40 in 2020, whereas they were RV-B4 and C53, RV-B79 and RV-C2, and RV-B14 and C15 in 2019, 2018, and 2017, respectively (Supplementary Fig. 2).

### Relationship between RV epidemic status and prevention measures

**Fig. 3:**
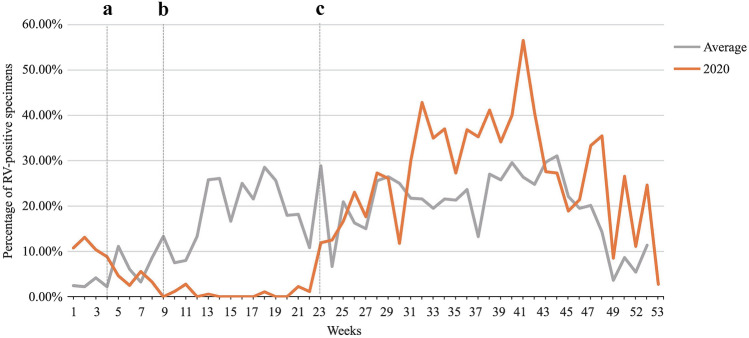
Weekly distribution of rhinoviruses (RVs) during Jan 2017–Dec 2020. **a** First-level public health emergency response was activated in Beijing, while a series of epidemic prevention measures had been implemented to restrict the outbreak of COVID-19; **b** the easing of the national lockdown, and some adults gradually were permitted to return to work; **c** the primary and secondary schools in Beijing were concurrently reopened. The gray line indicates the change in the weekly distribution of RV-positive rates from 2017 to 2019, and the red line indicates the change in the weekly distribution of RV-positive rates in 2020

In Beijing, prevention measures to restrict the outbreak of COVID-19, such as canceling mass gatherings, curbing population flow, extending the length of the Chinese New Year holiday, postponing the reopening of schools, making fewer trips outside, and mask wearing, have been implemented since January 24, 2020 (the 4th week). The results of the weekly positive rates of RV infections (Fig. 3) then showed the number of cases positive for RVs dropped rapidly from 8.82 to 0.00% and then remained < 3%, even when some adults were gradually permitted to return to work from February 23, 2020 (the 9th week). However, there was an increase in the number of RVs detected from the 23rd week (11.90%), gradually rising to 56.52% in the 41st week, which was far greater than that determined in the same period in the previous 3 years (26.36%, *P* = 0.005). Several noteworthy events occurred from June 1, 2020 (the 23rd week), including the concurrent reopening of primary and secondary schools in Beijing, followed by kindergartens from June 8, 2020 (the 24th week).

To reveal the correlation between the reopening of schools and kindergartens in Beijing and the surges of RV infection, all children were divided into two groups (Supplementary Fig. 3): younger (< 3 years) and older than three years (≥ 3 years). In group < 3 years, the positive rate of RV from the 23rd week to the 52nd week in 2020 (in average, 23.20%) was higher than that in previous 3 years (average in 2017–2019, 21.41%), which showed no significant difference (*P* = 0.363). In the group ≥ 3 years, the positive rate of RV between the 23rd and 52nd weeks in 2020 (in average, 30.2%) was significantly higher than that in the previous 3 years (in average, 17.0%, *P* < 0.001).

## Discussion

The results of the present study revealed that the detection rates of viral pathogens, such as the flu virus, RSV, RVs, and ADV, among children in Beijing were reduced greatly from February 2020 to May 2020 when the most stringent epidemic prevention measures were implemented in this city. In particular, the detection rates of RSV and flu dropped abruptly (by 92.17% and 94.67%, respectively), with an abrupt and early halt in the 2019–2020 flu season. Similar conclusions were obtained from several studies in which the local flu and RSV seasons ended early during this time [2, 13–15]. These results confirm that the series of preventative and control measures against SARS-CoV-2 were also effective in stopping the spread of other respiratory viruses.

Nevertheless, while most viral pathogens remained at a relatively low level, the positive rates of RVs increased from June 2020, reaching an apex in August 2020 (*P* = 0.005), which significantly exceeded that in the same period in 2017–2019. This change coincided with the reopening of the primary and secondary schools in the 23rd week of 2020 and subsequently the kindergartens from the 24th week of 2020 in Beijing. In a study of RV epidemiology in Southampton, UK from March 23 to September 20, 2020, the detection rates of RVs also were rebounded rapidly 2 weeks after the start of school [16]. Therefore, there was a correlation between the surges in RVs and the reopening of schools, which was supported by the positive rate of RV in group ≥ 3 years between the 23rd and 52nd weeks in 2020 (in average, 30.2%), significantly higher than that in the previous 3 years (in average, 17.0%, *P* < 0.001) in the study.

There were several explanations for the relationship between the surges in RVs and the reopening of schools. First, RV had relatively higher resistance to commonly used alcohol-based hand disinfectants [17]. After washing hands with soap and water, RV was detected from the left hand of 3/9 (33.3%) test persons and from the right hand of 1/9 (11.1%) test persons, whereas the virus was detected invariably from both hands (100%) after cleaning with an alcohol hand rub [17]. Second, although surgical masks can prevent people with symptoms from spreading HCoV and flu, substantial differences were not observed between detection of RV with or without face masks, both in respiratory droplets and in aerosols [18]. Third, it was difficult to maintain strict social distancing for children who returned to school. Fourth, the decreasing positive rates of other respiratory viruses in children in Beijing might have provided opportunities for the subsequent RV surges in 2020. It has been reported that after strict prevention and control measures are implemented, a shared ecological niche formed, leading to an increase in the number of people susceptible to viral infections [1, 19]. Therefore, it is easier for RVs to break through the preventive line in children, which subsequently resulted in high positive rates.

During the surges of RV hospital-based infection, the seasonality of RV species was not greatly affected, accompanied by significantly increased positivity rates of RV-A and RV-C in 2020. While no obvious circulation pattern was observed for each RV type, the dominant types were not detected in the consecutive years. Therefore, the RV types prevalent in 2020 were significantly different from those found in 2017–2019.

Our study had several limitations. Although we have discussed the unusual increase in RV infections, which might be associated with the reopening of schools, more data should be accumulated to confirm this association. Following the relaxation of the prevention and control measures and the gradual normalization of social life, the epidemiological characteristics of respiratory viral pathogens, especially RVs, also should be evaluated more intensely [20].

In summary, our results indicated that the prevention and control strategies for the COVID-19 pandemic were not only effective at blocking the spread of SARS-CoV-2 but also had a significant impact on decreasing the spread of respiratory viruses, especially RSV and flu, with an absence of flu and RSV seasons in winter 2020. We also observed RV infection surges from the 23rd week of 2020, which might be explained by the reopening of schools and kindergartens.

## Supplementary Information

Below is the link to the electronic supplementary material.

Supplementary Fig. 1. Phylogenetic tree constructed using MEGA version 6.0 software to identify the types of rhinovirus (RV)-positive specimens. The blue branches represent RV-A, red branches represent RV-B, and orange branches represent RV-C. Each outermost colored band indicates one RV type

Supplementary Fig. 2. Schematic showing the monthly distributions of the different rhinovirus (RV) types. A: Distribution of each type of RV-A; B: distribution of each type of RV-B; C: distribution of each type of RV-C

Supplementary Fig. 3. Weekly distribution of rhinoviruses (RVs) of different ages during Jan 2017 to Dec 2020. A:The percentage of RVs-positive specimens from children younger than 3 years (<3y) in 2020 (red curve) compared with the average ones in 2017-2019 (grey curve). B: The percentage of RVs-positive specimens from children older than three years (≥3y) in 2020 (red curve) compared with the average ones in 2017-2019 (grey curve). a. the First-level public health emergency response was activated in Beijing, while a series of epidemic prevention measures had been implemented to restrict the outbreak of COVID-19; b. the easing of the national lockdown, and some adults were gradually permitted to return to work; c. the primary and secondary schools in Beijing were concurrently reopened

## Data Availability

The datasets generated during and/or analysed during the current study are available from the corresponding author on reasonable request.
