# Supplementary figures and images for: Surges of hospital-based rhinovirus infection during the 2020 coronavirus disease-19 (COVID-19) pandemic in Beijing, China
Source: World J Pediatr. 2021 Oct 28;17(6):590–6. doi: 10.1007/s12519-021-00477-2 (PMC8552974; doi:10.1007/s12519-021-00477-2)

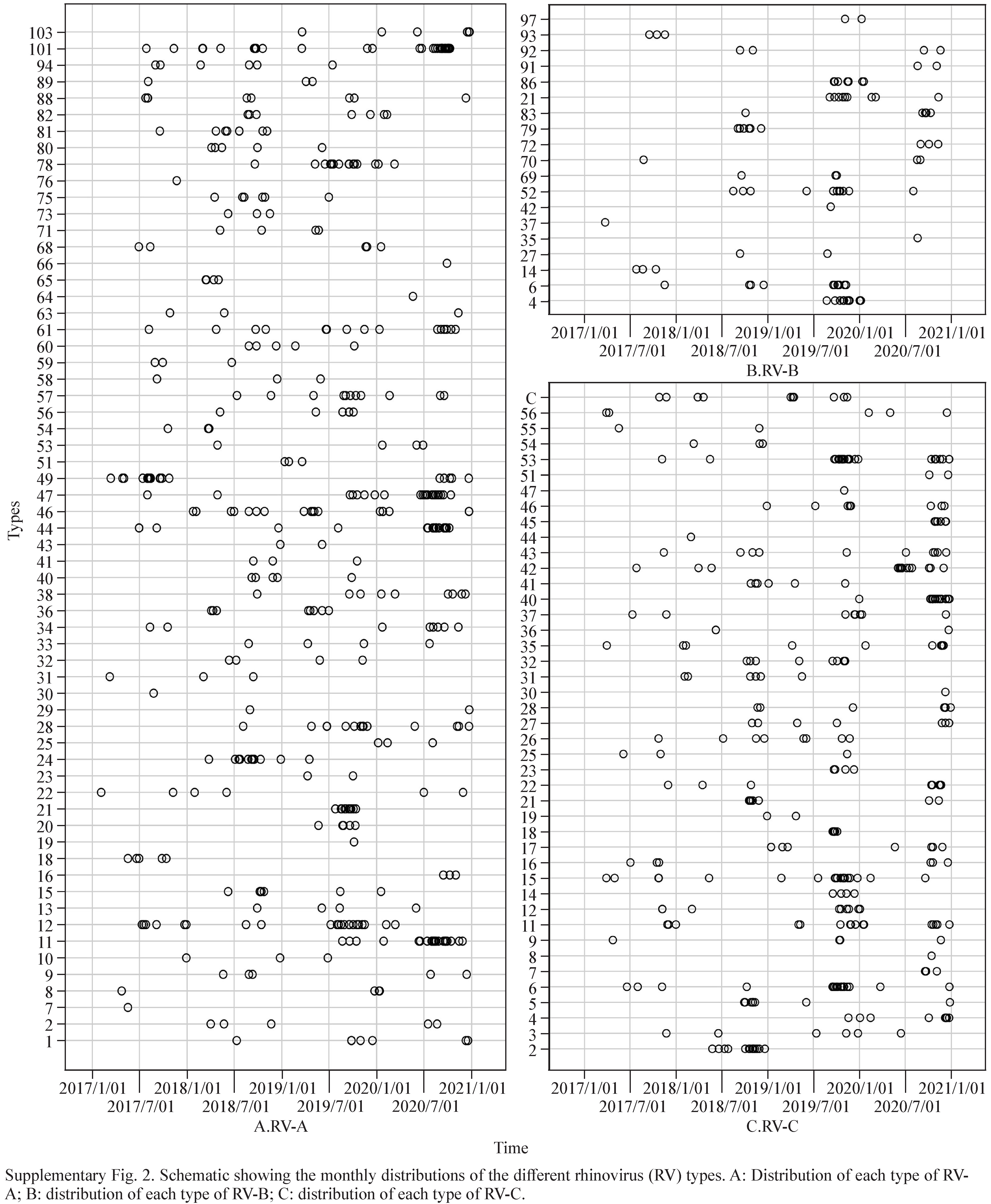

Supplement: Supplementary file 1 — Supplementary Fig. 1. Phylogenetic tree constructed using MEGA version 6.0 software to identify the types of rhinovirus (RV)-positive specimens. The blue branches represent RV-A, red branches represent RV-B, and orange branches represent RV-C. Each outermost colored band indicates one RV type [file 12519_2021_477_MOESM1_ESM.tif]

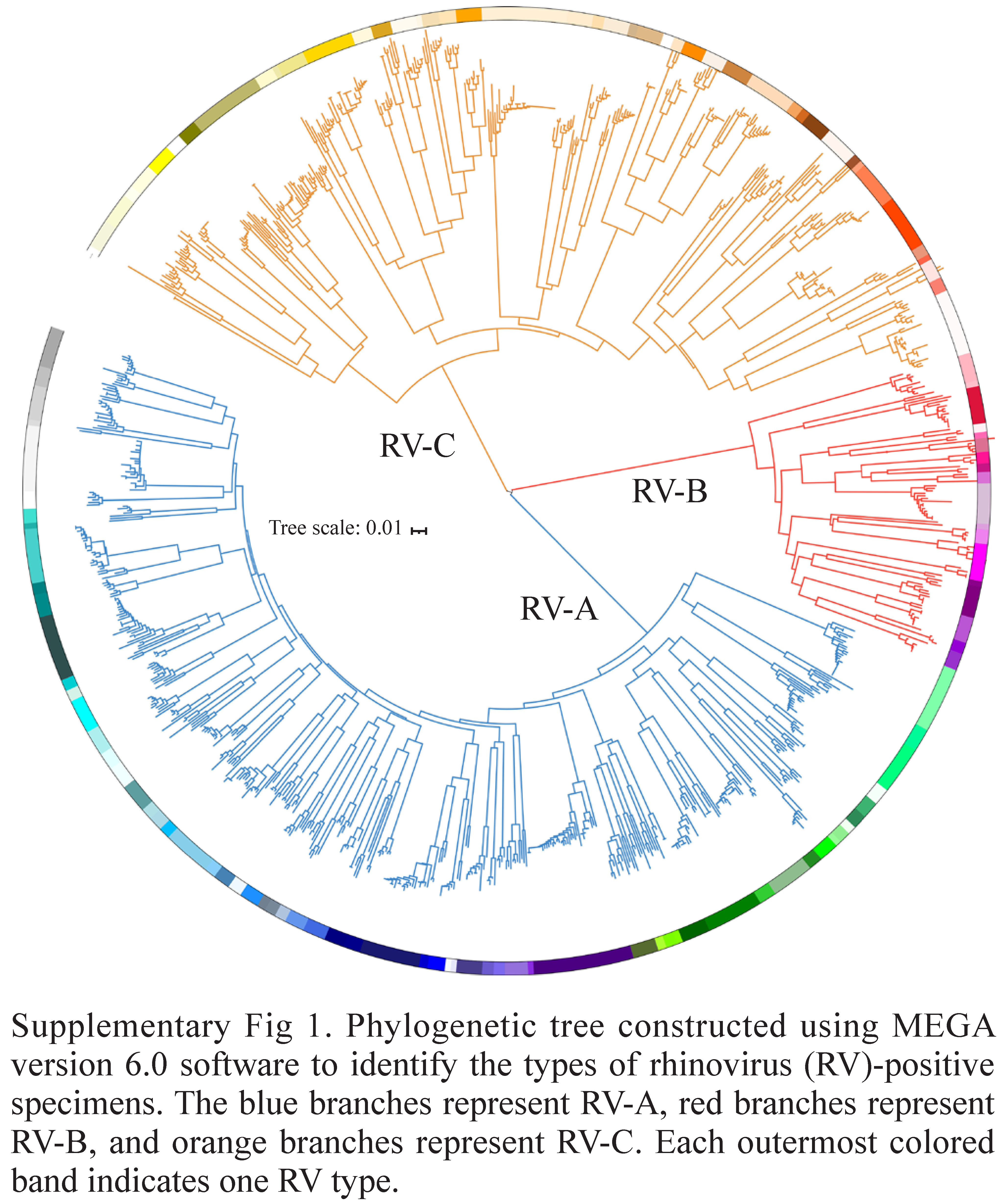

Supplement: Supplementary file 3 — Supplementary Fig. 3. Weekly distribution of rhinoviruses (RVs) of different ages during Jan 2017 to Dec 2020. A:The percentage of RVs-positive specimens from children younger than 3 years (<3y) in 2020 (red curve) compared with the average ones in 2017-2019 (grey curve). B: The percentage of RVs-positive specimens from children older than three years (≥3y) in 2020 (red curve) compared with the average ones in 2017-2019 (grey curve). a. the First-level public health emergency response was activated in Beijing, while a series of epidemic prevention measures had been implemented to restrict the outbreak of COVID-19; b. the easing of the national lockdown, and some adults were gradually permitted to return to work; c. the primary and secondary schools in Beijing were concurrently reopened [file 12519_2021_477_MOESM3_ESM.tif]
